# Health Data Nexus: an open data platform for AI research and education in medicine

**DOI:** 10.1093/gigascience/giaf050

**Published:** 2025-06-03

**Authors:** January Adams, Rafal Cymerys, Karol Szuster, Daniel Hekman, Zoryana Salo, Rutvik Solanki, Muhammad Mamdani, Alistair Johnson, Katarzyna Ryniak, Tom Pollard, David Rotenberg, Benjamin Haibe-Kains

**Affiliations:** Temerty Centre for Artificial Intelligence Research and Education in Medicine, Temerty Faculty of Medicine, Department of Laboratory Medicine and Pathobiology, University of Toronto, Toronto, Ontario M5S 1A8, Canada; Upside Lab, 10117 Berlin, Germany; Upside Lab, 10117 Berlin, Germany; Upside Lab, 10117 Berlin, Germany; Temerty Centre for Artificial Intelligence Research and Education in Medicine, Temerty Faculty of Medicine, Department of Laboratory Medicine and Pathobiology, University of Toronto, Toronto, Ontario M5S 1A8, Canada; Temerty Centre for Artificial Intelligence Research and Education in Medicine, Temerty Faculty of Medicine, Department of Laboratory Medicine and Pathobiology, University of Toronto, Toronto, Ontario M5S 1A8, Canada; Unity Health, Toronto, Ontario M6R 1B5, Canada; Glowyr, Hebden Bridge HX7 8AA, UK; Upside Lab, 10117 Berlin, Germany; Laboratory for Computational Physiology, Massachusetts Institute of Technology, Massachusetts 02139, USA; The Centre for Addiction and Mental Health, Toronto, Ontario M6J 1H4, Canada; Princess Margaret Cancer Centre, University Health Network, Toronto, Ontario M5G 2C4, Canada

**Keywords:** data science, artificial intelligence, medicine, data platform

## Abstract

We outline the development of the Health Data Nexus, a data platform that enables data storage and access management with a cloud-based computational environment. We describe the importance of this secure platform in an evolving public-sector research landscape that utilizes significant quantities of data, particularly clinical data acquired from health systems, as well as the importance of providing meaningful benefits for three targeted user groups: data providers, researchers, and educators. We then describe the implementation of governance practices, technical standards, and data security, and the privacy protections needed to build this platform, as well as example use-cases highlighting the strengths of the platform in facilitating dataset acquisition, novel research, and hosting educational courses, workshops, and datathons. Finally, we discuss the key principles that informed the platform's development, highlighting the importance of flexible uses, collaborative development, and open-source science.

## Background

The fast-growing field of machine learning (ML) and artificial intelligence (AI) holds transformative promise for the field of medicine, offering prospects for enhancing patient outcomes and improving healthcare delivery. These technologies are pivotal in driving advances across a spectrum of applications, including the early detection of emergent medical conditions, the development of diagnostic and prognostic tests, and the acceleration of drug discovery and development.

The transformative potential of AI in healthcare depends on the availability and accessibility of large, high-quality datasets. The healthcare sector is a prolific generator of data, yet this information is often siloed, dispersed across various entities, and mired in issues of accessibility for research purposes. Moreover, the data landscape in healthcare is characterized by a lack of uniform standards [1], compounded by inadequate documentation, which collectively pose significant challenges to the effective use of these data in ML/AI applications.

To harness the full transformative potential of ML/AI in medicine, these barriers must be addressed, thereby fostering an environment in which data sharing is streamlined, standardized, and conducted in a manner that upholds the high standards of data privacy and security. Both comprehensive, well-curated datasets and advanced ML/AI methodologies are crucial to unlocking innovative healthcare solutions that are precise, predictive, and patient-centric.

Although many healthcare and health research organizations house data for secondary uses such as research, access to these datasets can be cumbersome because they are usually secured and only made available to researchers upon approval of a research plan and in collaboration with representatives of the organization. Once access is approved, researchers are often required to use their own external computing resources to analyze the data. Although this process minimizes the risk of data misuse and leakage, it is lengthy and presents a significant barrier to health innovation through research.

Other data platforms have taken a more accessible approach to data sharing. One example is PhysioNet [2], a data-sharing platform created and maintained by the Laboratory for Computational Physiology at the Massachusetts Institute of Technology in the United States. PhysioNet provides a platform for users to share datasets and software and includes a workflow for approving and credentialing potential data users, who can then download data to work on their own projects. The entire platform is built as an open-source project, allowing other researchers and institutions to make use of the same framework. This platform exists within an US regulatory environment where different restrictions apply, potentially limiting the access to some datasets. Other platforms such as Nightingale Open Science [3] use a similar framework for development.

Given the complexity and sensitivity of health data, striking the right balance between openness, ease-of-use, scalability, and security remains an open challenge. Openness and ease-of-use are crucial to ensure that data can be accessed in an equitable and inclusive manner for a diverse group of users. As new technologies such as electronic medical records and sequencing are adopted, the amount and complexity of health data increases, calling for the design of digital platforms that are scalable from the perspective of computational resources. Further, many health datasets are highly sensitive and may remain so after deidentification, requiring the data platform to be secure and able to accommodate the potential requirements for access restrictions. Such restrictions can take the form of an approval from an institutional review board (IRB) or research ethics board (REB), a data-usage agreement, or a review through a data-access committee. Recognizing that generating high-quality health data requires expertise and resources, it is also essential that a platform offer strong incentives for data generators to contribute their datasets, creating a virtuous cycle between users and data providers.

To strike a balance between data security, usability, and streamlined access, we developed the Health Data Nexus (HDN) [4] within the Temerty Centre for Artificial Intelligence Research and Education in Medicine (T-CAIREM) [5]. T-CAIREM is a research centre at the University of Toronto. HDN was developed as a platform for both the storage and analysis of deidentified health data for the academic research and education communities in Canada. Building upon the scalable Google Cloud Platform (GCP) and PhysioNet’s user-friendly interface, we have implemented multiple options for data access and use. This makes HDN an open-source and well-balanced platform for research and education. Here, we describe the key characteristics of the HDN platform, its implementation, and its usage to transform research and education for AI in medicine.

## Motivation

**Figure 1:**
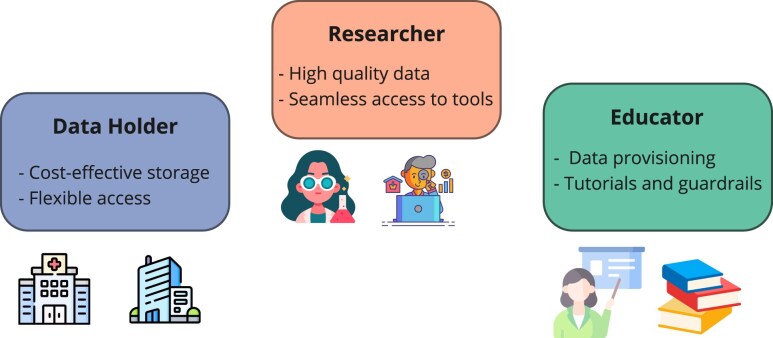
Summary of the three main user groups and their requirements on the platform.

Acquiring real-world health data traditionally involves hosting data directly from healthcare systems; however, this approach frequently encounters significant barriers due to data security and privacy. Consequently, data providers must impose stringent restrictions on data availability and implement rigorous limitations on access, often complicating and slowing research initiatives. An alternative solution involves the use of synthetic data, although this approach introduces its own set of challenges, notably in preserving data realism and ensuring applicability of analytical findings to actual clinical scenarios. Recognizing these complexities, we developed HDN at the intersection of data security, accessibility, and usability with three principal objectives—dataset curation, research enablement, and educational support—each tailored to meet distinct user-group requirements (Fig. 1). Below, we describe these core goals, the specific user groups they represent, and the technical and governance challenges addressed to achieve them.

### Data holders

Data holders seek simple, cost-effective, and secure data storage tailored to their individual needs. Whether they aim to make their data broadly available to a large number of potential users or only to a single lab or small group of trusted researchers, this requires a streamlined process. This process should include simple and understandable rules around governance and data hosting, as well as easy access to a data steward to assist with the process. Providers may also need flexibility around approving different levels of access to various potential users, allowing them to maintain control over their data while accommodating the needs of researchers.

### Researchers

Researchers require access to high-quality data, along with the tools and support needed to conduct their analyses. They require the ability to seamlessly load data into a safe and secure research environment equipped with packages, essential software, and computing resources. Researchers also benefit from access to learning resources, such as user-contributed tutorials and methods or comprehensive metadata to describe the dataset. Helpful built-in support mechanisms can enable higher-quality and timely completion of their projects; examples of these supports include access to knowledgeable support staff or a community of researchers to resolve research questions.

### Educators

Educators seek to distribute data using the platform, either to their students or to the attendees of a workshop or datathon. Educators benefit from a simple and streamlined method to provide access to datasets, complemented by tutorials that can be integrated into data environments as a starting point. They focus on provisioning data access to many users, emphasizing simplicity and ease over powerful computing resources. Educators may also benefit from mechanisms to prevent students or trainees from using more computing resources than required, preventing users from incurring greater financial costs.

## Implementation

**Figure 2:**
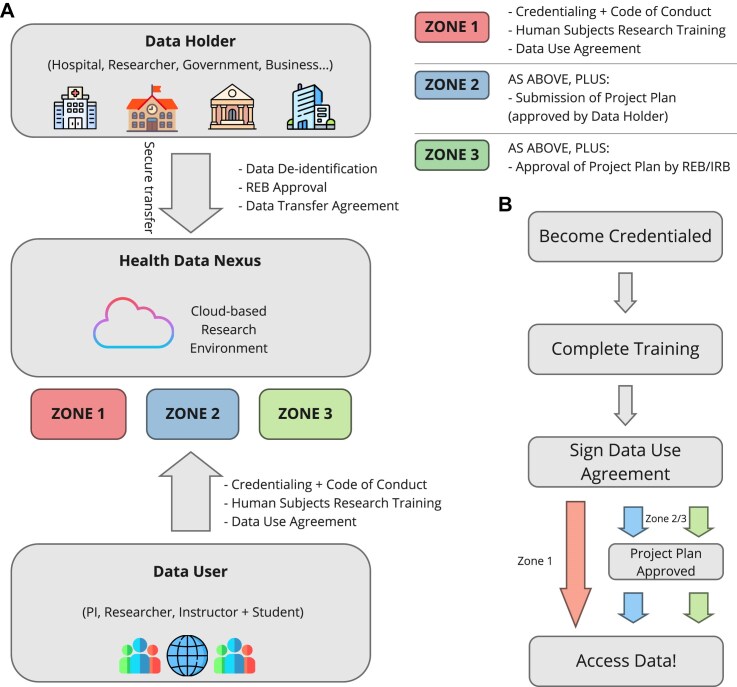
(A) An overview of the workflow for uploading data to the platform (for data holders) and accessing data (for data users), highlighting the different zones of access. (B) Details of the process for users to access data.

**Figure 3:**
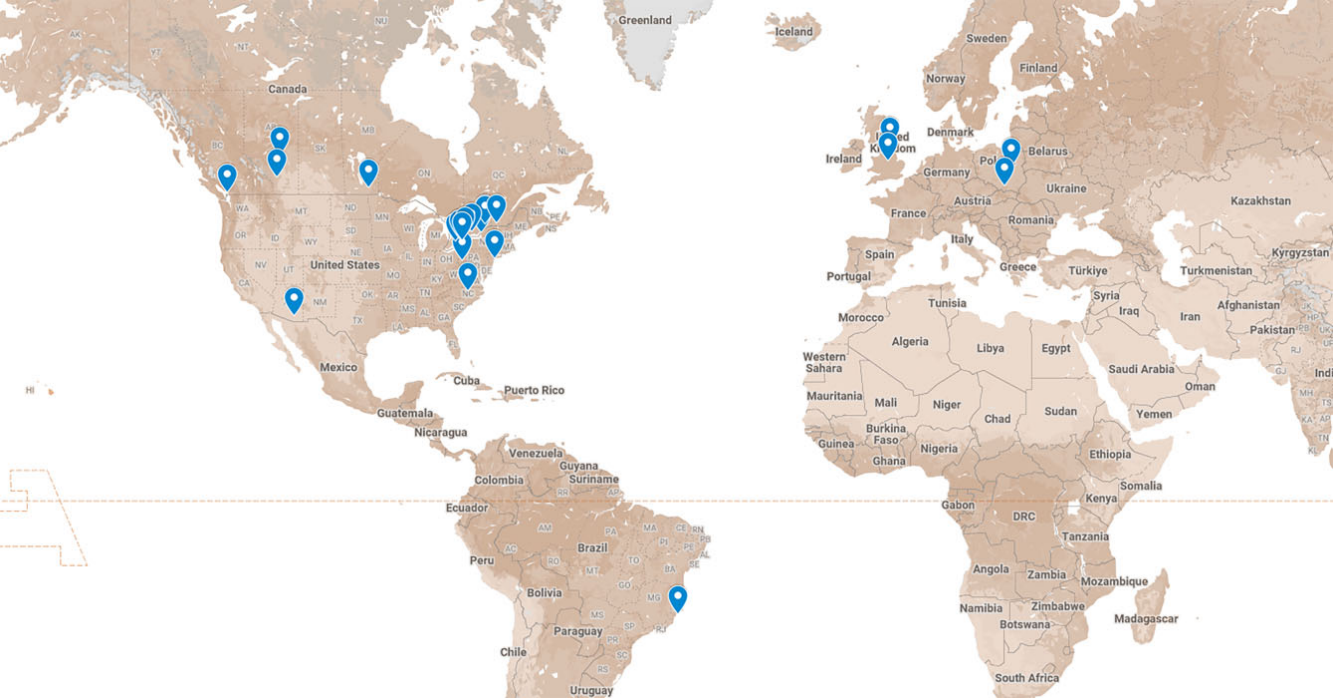
A map of universities in which at least one user with an associated university email address has accessed the platform.

Based on the user-specific needs, we outline the requirements for the HDN platform, including both governance requirements for data access (Fig. 2) and technical requirements around data storage and the associated analysis platform (Fig. 3).

### User interface

The user-facing interface of the HDN platform has been designed to meet the needs of its user groups by being specific, social, and streamlined. We define a platform as specific if it is designed to meet the direct needs of users—in our case, the platform should be focused on health data, and use familiar tools and resources to do so. The front-end of the HDN platform is based on the PhysioNet platform, which provides the credentialing model and brings the capability to manage datasets, data users, and their access to the datasets. PhysioNet’s interface was chosen partly due to its familiarity to users—many health researchers will be familiar with the interface required to access the very popular MIMIC-IV dataset. In addition, the PhysioNet structure was designed to handle multimodal data, including electrophysiological signals, images, tabular data, and free text, including combinations of these sources within a single dataset.

We also designed the user interface to be social, primarily in that it is open source and facilitates collaboration within the wider scientific community. The source code of PhysioNet, which underpins HDN, is accessible under the BSD 3-Clause license. During development of HDN, its code underwent significant modifications to accommodate the hosting of various data repositories across different scientific domains. We adjusted the platform to follow 12-factor app principles [6], allowing it to be deployed via the Google Kubernetes Engine, used Google Cloud Storage for storing datasets, and introduced a fully automated continuous integration and continuous delivery pipeline. These adjustments all update HDN to adapt to a modern technical infrastructure. These features were implemented back into the PhysioNet repository, allowing others to make use of them in the future and furthering our commitment to development through open science.

Finally, we aim for the user interface to provide a simple approach to managing dataset access. HDN supports curation and review processes for dataset publication and leverages Google Cloud Storage for internal data storage and managing dataset access permissions on the cloud level. Additionally, the interface facilitates user access management, overseeing the credentialing process and setting access prerequisites for datasets, such as mandatory training completion. To expedite credentialing, the platform enables account creation via eduGAIN sign-in, which verifies university affiliations. Moreover, the front-end supports event management, particularly for datathons, by offering features such as temporary dataset access. This functionality is crucial for ensuring data-use compliance during specific events.

### Computational infrastructure

The computational infrastructure of the platform has been designed to be secure, scalable, and streamlined. Security on the platform is crucial, not only to protect the information of users but also because of the sensitive nature of health data stored on HDN. Using a public cloud provider also makes it possible to control the region where the data are stored and processed in order to comply with local regulations. Additional controls have been implemented which prevent data from being directly downloaded or removed from a secure Google Cloud bucket. Using a public cloud provider also allows control over the region where the data are stored and processed to comply with local regulations.

We leveraged the cloud to make HDN highly scalable, allowing users to access their desired level of computing power. Due to cloud computing, HDN is not bound by the hardware resources of the academic institution and can scale on-demand to serve the needs of researchers. Using a cloud platform also makes it easier to control billing for resources used. We developed an architecture that allows for use of external billing methods, vastly simplifying the operational overhead at TCAIREM’s end and allows for a self-service process of creating research environments.

Finally, we developed a streamlined computational infrastructure, allowing for a clear distribution of responsibilities and standardized tools for the reuse of well-established technological stacks and frameworks. For users, the back-end provides templates for research environments with the desired configuration, with an ability to launch Jupyter- and RStudio-based environments and connect them to cloud services. Other analytics tools and research environments can be configured for access if needed. Upon a user’s request for a new research environment, the back-end handles the allocation of necessary cloud resources, the deployment of specified tools, and the authorization to datasets through appropriate Google Cloud Platform policies. It then simplifies dataset access by presenting them through the familiar file system provided by the Cloud Storage FUSE tool. The back-end orchestrates administrative actions within the Google Cloud Platform, streamlining the creation of Cloud Identity for users and managing their billing account set-ups. It supports custom billing arrangements, such as sharing a billing account among multiple users from the same organization.

The flexibility provided by the cloud infrastructure is also important for platform development. New features, types of environments, and tools can be added dynamically as needed. The back-end also orchestrates processes such as managing data access using cloud infrastructure, provisioning environments, and controlling billing (Supplementary Figs. 1 and 2).

### Governance model

HDN is built under the context of Canadian and Ontario privacy legislation and research guidelines, which inform the governance model of the platform. Relevant documents include Ontario's Personal Health Information Protection Act (PHIPA) (2004) [7] and the Tri-Council Policy Statement on Ethical Conduct for Research Involving Humans (2022) [8]. These provisions correspond with HIPAA (1996) [9] in the United States, whose guidelines have also been followed in the development of platform governance. The governance model of HDN has been reviewed by a privacy impact assessment conducted by a highly specialized legal firm. In addition, the technical capabilities and security have been subject to a threat risk assessment.

### Data deidentification

To avoid potential complications related to working with personal health information under PHIPA, HDN exclusively works with deidentified health data. The governance process begins with T-CAIREM signing a data transfer agreement with an interested party (the data holder; Fig. 2A). The data holder is responsible for deidentifying the data to an appropriate standard (as determined by T-CAIREM and informed by the Information and Privacy Commissioner of Ontario [10]) and for providing the dataset to T-CAIREM after any appropriate preprocessing. The data holder will also provide associated documentation and metadata needed to understand and use the data and will upload the dataset to HDN with the help of the T-CAIREM data steward.

### User credentials

Before logging into the platform, potential users can view the collection of datasets available on the platform. They can then access the dataset by logging into the platform by single sign-on through their university or academic institution. Once logged in, a user can become an authorized dataset user for a particular dataset by doing the following (Fig. 2B):

1. Becoming credentialed on the platform by submitting personal information and a reference.
2. Completing any required training associated with the dataset (at minimum TCPS 2: CORE-2022 training associated with the Tri-Council Policy Statement on Ethical Conduct for Research Involving Humans) [8].
3. Signing a data use agreement, unique to each dataset and approved by both T-CAIREM and the data holder.

An authorized dataset user is given full access to the requested dataset, and can access it by creating a cloud identity and a research environment for the purposes of analysis.

### Data access levels

All described governance provisions apply to all datasets on the platform; however, data holders may wish to provide additional protection for particularly sensitive datasets. To this end, we have introduced three levels of data access (Fig. 2A). The first level of access (“Zone 1”) includes all requirements outlined above. The second level of access (“Zone 2”) includes all requirements outlined above, in addition to requiring a research plan to be submitted by the interested user, which must be approved by the data holder. The third and final level of access (“Zone 3”) includes all requirements outlined above, in addition to requiring a research plan which must be approved not only by the data holder but also by an appropriate REB. Higher zones of access may be appropriate when dealing with potentially sensitive data, or when data holders want community stakeholders to play a role in determining dataset access.

### Comparison with other data platforms

Although other prominent data platforms exist, the HDN platform displays a unique combination of features (Table 1). This list of platforms is non-exhaustive; other platforms exist in the literature as a point of comparison [11–13]. By comparison, we consider data platforms such as Kaggle [14] and ICES [15].

**Table 1:** A comparison of the offerings on different data platforms.

Platform	Available Data	Analysis Options	Security	Accessibility
HDN	Approximately 10 curated and well-documented datasets covering topics related to medicine and health	Data can be accessed and analyzed in a cloud computing environment after a brief onboarding process	Data cannot be directly downloaded and are subject to data-transfer and data-use agreements, allowing for secure sharing of patient data	Account registration, credentialing, and training can be completed within a span of hours, giving users same-day access to datasets
Kaggle [14]	Almost 450,000 datasets across a wide variety of fields; datasets can be uploaded by any user with minimal curation	Data can be directly downloaded and analyzed, or uploaded into an online analysis platform	Data security is minimal, and the platform is not suited for hosting potentially sensitive data	Data can be immediately downloaded and accessed
PhysioNet [2]	Over 350 datasets, curated for the platform, in addition to software and resources	Data can be downloaded directly or accessed in Google BigQuery	Data are curated and reviewed before hosting, but direct downloads mean data are not protected against reidentification or linkage attacks	As with HDN, most datasets can be accessed with same-day approval
ICES (formerly Institute for Clinical Evaluative Sciences) [15]	Over 100 datasets, primarily related to health services operation	Data are only accessible upon review of a project plan approved by an ICES scientist, available in a limited analysis environment.	ICES is a prescribed entity per PHIPA, meaning it can safely and securely host data (including sensitive data which include identifiable information)	An in-depth data access form is required, taking time to put together as well as time to review

## Case studies

Early results demonstrate the capacity of HDN to facilitate data acquisition and educational opportunities.

### Data acquisition

One of the key value adds of HDN has been providing data storage at no cost. This provides an effective way to engage data holders and spread interest in the platform.

As of May 2025, HDN hosts 9 datasets comprising structured tabular data, images, and population-level health data (Table 2). The St Michael's Hospital General Internal Medicine (GIM) dataset [16] is the flagship dataset for the platform. This dataset provides a wide range of patient information on 14,000 patients over 22,000 visits, including outcomes, tests, medication information, and demographics, and provides a gold standard of tabular data on the platform. Another key dataset is the Cervical Spine CT Scan (CSpine) Dataset, also from St Michael’s Hospital [17]. This is the largest imaging dataset on the platform, containing a total of over 1,000 computed tomography (CT) scans. This sets a precedent for HDN hosting a wide variety of datasets of different modalities.

**Table 2:** An overview of the datasets available on HDN.

Dataset	Abbrev	Source	Summary
General Internal Medicine [16]	GIM	St Michael's Hospital	In-patient data drawn from EHR, Admit Discharge Transfer System, and Medication Administration Check System on 22,000 patient encounters
Cervical Spine CT Scan [17]	CSpine	St Michael's Hospital	1,000+ merged CT scans in DICOM format
COVID-19 In-Patient Datasets [18]	THP-C19	Trillium Health Partners	Demographic, clinical, and outcome-related data on 500+ patients
Canadian Heart Health Database [19]	CHHDB	Canadian Heart Health Initiative	Country-wide survey data on 23,000 entries across all 10 Canadian provinces
Trial Files [20]	–	Mount Sinai Hospital	Summary of the results of randomized control trials published in clinical journals
Canadian Community Health Survey [21]	CCHS	Statistics Canada	Nationwide survey on health status, healthcare utilization, and health determinants
COVID-19 Epidemiology and Vaccination Data [22]	–	COVID-19 Canada Open Data Working Group	Epidemiological dataset for COVID-19
Sleep Laboratory Dataset [23]	–	Sunnybrook Research Institute	Data from Sunnybrook Sleep Laboratory that include deidentified raw overnight signals, scored sleep metrics, sleep and health questionnaires, and medications/medical history
Bridge2AI-Voice Dataset [24]	B2AI	Multi-site (based in University of South Florida)	A dataset of voice recordings and metadata to enable the development, benchmarking, and validation of clinically applicable machine-learning models for diagnosing a wide range of health conditions.

The other datasets on the platform are the COVID in-patient dataset from Trillium Health Partners [18], the Canadian Heart Health Database [19], Trial Files for Randomized Control Trials on Large Language Models [20], the Canadian Community Health Survey [21], COVID-19 Epidemiology and Vaccination data [22], the Sleep Laboratory Dataset [23], and the Bridge2AI-Voice Dataset [24].

### User diversity

**Figure 4:**
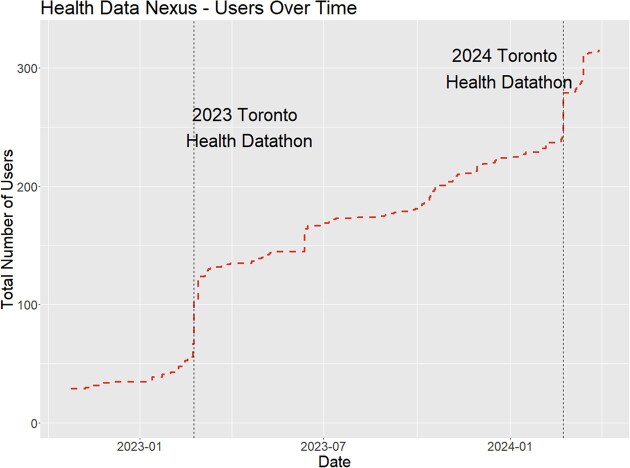
Number of users on the platform over time, highlighting the increase in users corresponding with major events (the 2023 and 2024 Toronto Health Datathons). The GIM dataset was first uploaded on 21 October 2022, and the CSpine dataset was first uploaded on 10 February 2023.

**Figure 5:**
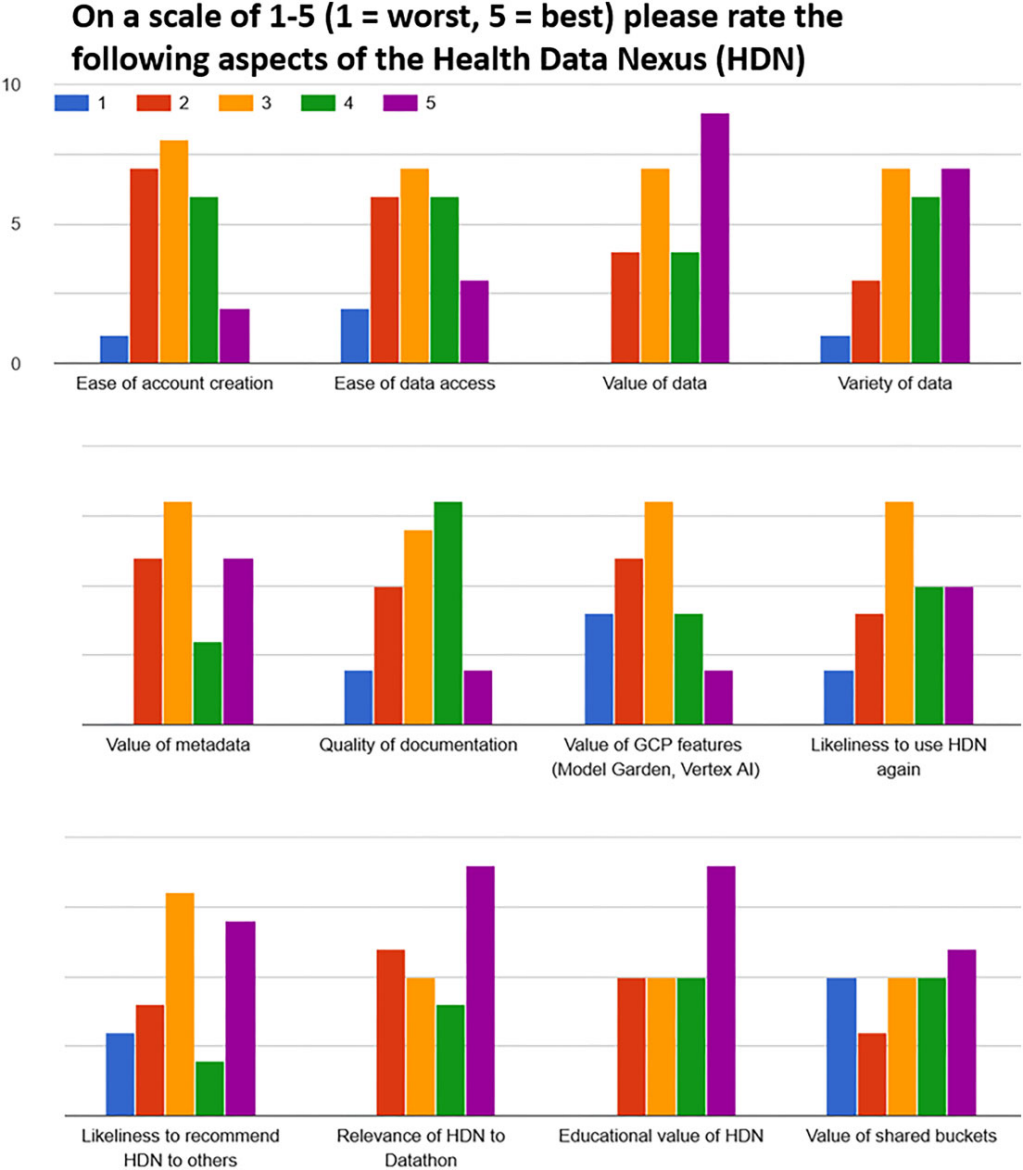
Responses to a post-event survey from the Toronto Health Datathon 2025 on the value of different components of HDN (*n* = 24 responses). Questions included accessibility and value of both the datasets and supplementary features.

HDN attracts a wide range of users from 5 different countries (Fig. 4). Users are primarily from Canadian universities in the province of Ontario (93%) with the remaining proportion (7%) being from the United States, Brazil, Poland, the United Kingdom, and the rest of Canada. The growth of the user base over time (Fig. 5) reflects the inclusion of new datasets and major events centered on HDN, notably the two Toronto Health Datathons hosted using the platform.

### Education

HDN has seen the greatest success as a tool for educators, providing an easy way to provide students and trainees with access to high-quality medical datasets. One of the first and ongoing demonstrations of this use case has been through a professor in biomechanical engineering. Over 2 years, this professor has run short-term projects with students that involve providing access to the GIM [16], COVID-19 [18], and Canada Heart Health [19] datasets and then developing small-group projects. Access to real medical data has been helpful for introducing students to some of the challenges of working with real-world data, including inconsistencies, preanalysis, bias, and class imbalances that are present in real-world health datasets.

HDN has also been used for workshops, such as the VADA Summer Schools workshop held between the University of Victoria and the University of Manitoba [25]. This week-long workshop involved a 2-day group project for students to get access to datasets and test out analytics. The ease of onboarding has been facilitated through an events workflow on the platform, which provides a large number of users with an easy way to become credentialed. In addition, the use of shared billing accounts provided by the course administrator or professor dispenses with the requirement for each student to use their own billing information, which may be an equity concern. We continue to explore new ways of providing ease of access and providing a fulfilling educational experience for users, such as promoting Google Research Credits [26] for educators and workshop organizers.

### Datathons

The Toronto Health Datathon has been held for 3 years, in 2023, 2024, and 2025. This event brings students, trainees, data scientists, and healthcare professionals together for a 2-day event. Participants join groups to tackle a problem related to 1 of 3 available datasets. In both the 2023 and 2024 datathons, datasets have included the GIM in-patient tabular data, COVID-19 patient data, and Cervical Spine CT Scan imaging data. In the 2025 datathon, datasets included the GIM dataset, the Sleep Study dataset, and the Bridge2AI Voice dataset.

Datathons have provided valuable feedback and suggested improvements to HDN. Between the 2023 and 2024 datathons, improvements to the platforms included providing cloud storage for group members to share code and resources, implementing shared billing accounts, and refining and improving the stability of the platform. In a post-event feedback survey following the 2024 datathon, 19 of 25 participants (76%) agreed or strongly agreed that the HDN platform met their expectations. Constructive feedback on the platform included performance issues and providing higher-power computing resources, including access to more powerful GPUs and tools associated with Google’s Vertex AI. This feedback will be incorporated as we continue to develop the platform.

The 2025 Datathon placed an additional focus on evaluation feedback. We have included a summary of the feedback in Fig. 5 and highlight a few key results:

- Participants highlighted the value and variety of the data on the platform, including their educational relevance.
- Some participants found that some of the supplemental features of the platform, such as the option to attach a shared bucket to a workspace for sharing code and results, were of limited value in their current state.
- Participants were mixed on the ease of account creation.

## Discussion

### Collaboration between industry and academic partners

The HDN initiative emerged through a strategic collaboration between academia and industry, combining the resources and expertise of both sectors. Academic partners, including the University of Toronto and Massachusetts Institute of Technology, contributed invaluable domain knowledge, subject expertise, and deep insights into the PhysioNet platform. Conversely, the industry partner played a pivotal role by providing software development expertise, overseeing the development cycle, and implementing best practices in executing technology projects.

Although the collaboration between academic and industry partners—often referred to as academia-to-business or university–industry collaborations—holds significant potential for generating valuable results, it is crucial to identify and address common challenges associated with it. Over the course of the project, the team examined, assessed, and adjusted various aspects, including:

1. Understanding of the common vision;
2. Alignment of short- and long-term goals;
3. Effectiveness of collaboration and software development processes;
4. Quality and timely delivery of outcomes.

These areas were identified as major challenges in university–industry collaborations [27] due to often differing contexts and realities that underlie project motivation (research and curiosity vs delivery and revenue), planning (discovery, research, open timeline vs time and budget constraints), and execution in university–industry partnerships.

To address the challenges commonly encountered, the teams adhered to the principles of agile software development [28]. This methodology underscores the importance of moving towards a common goal through iterative processes, featuring short feedback loops, close stakeholder collaboration, and adaptability to evolving requirements. Embracing this approach proved fundamental throughout the development phase, facilitating prompt and well-informed decision-making, particularly evident in the assessment of the capabilities of various cloud platforms.

Despite the distributed nature of the team, collaboration remained close throughout the project. To ensure timely and frequent feedback the team engaged in week-long development “sprints.” These sprints incorporated weekly meetings for work review, discussion, and planning, alongside daily synchronous and asynchronous communication to track progress. Throughout the project’s duration, both teams demonstrated responsiveness and active participation, contributing to its success.

Next to making sure the teams can work together towards a goal, an alignment in understanding and approach towards commercial aspects is needed. Just as in any software development project involving multiple stakeholders, it is crucial to establish consensus on critical factors such as intellectual property rights ownership and transfer, commercialization and licensing strategies, resource allocation, risk-management protocols, deliverables acceptance criteria, payment agreements, and tracking mechanisms.

### User curation

An important part of developing HDN as a viable and well-used platform is curating both the selection of datasets and the users on the platform.

It is important to have a wide collection of datasets that cross a variety of subjects and data types. HDN currently includes tabular data of patient records (from both general internal medicine and COVID-19 wards) and CT scan imaging data, with plans to expand to include videos, different types of imaging data, genomics data, and deidentified free text, in addition to a wider variety of imaging data. Acquiring these datasets relies on relationship building and demonstrating the value of HDN to interested data holders.

Curating an active and engaged platform user base also relies heavily on building relationships with scientific communities, reaching out via communications and social media, and developing a set of high-value datasets that provide worthwhile value to users. Effective documentation (described below) and streamlined processes for providing support to users are essential for ensuring adoption of the platform.

### Documentation

Provision of useful and accurate documentation for users is a key reason for the success of HDN. Each dataset on HDN is provided with a data page, adapted from the structure used for PhysioNet, that includes essential information about the dataset. Including background information, methods used in dataset development, usage notes, and a detailed description. This provides users with the essential information needed for their analysis.

Additionally, HDN uses a system of supplemental documentation adapted from the Divio documentation system [29]. This system uses 4 complementary types of documentation. The Data Dictionary provides detailed information about every field in the data tables included under a dataset. The Explanation and Backgrounds provides additional context on how the dataset was created and for what purpose. The Tutorials provide in-depth guides on performing basic analysis using the dataset. The Getting Started guide connects users with the resources they need to load up the analysis for their first time using the data.

The supplemental documentation is hosted on an external dashboard linked from the data page. The combination of these resources gives users the full set of information they need to work with the data.

Finally, usability is key to ensure that interested researchers, educators, and students have a successful experience with the platform. In addition to the resources provided, a variety of information helps inform new users to the platform, including instructional content on the website and a series of tutorial videos.

### Future directions

Continued improvement of HDN is a key priority moving forward. Some suggested features include new analysis environments, tools, and research software, and additional computing resources and Google Cloud tools for data analysis. These suggestions reflect the addition of new datasets in a wide variety of fields, including imaging and video analysis, free-text data, genomics data, and electrical and physiological signals. In particular, development will include features for sharing relevant code and models among users of a dataset. This will allow for improved educational opportunities among users and the development of a shared body of knowledge. To improve ease of use, development will also include implementing standards such as the Fast Healthcare Interoperability Resources (FHIR) [30] for new and existing datasets on the platform.

Development plans also include involving new partners in the progression of HDN. In addition to attracting new researchers, educators, and data holders, we plan to engage industry. Corporate datathons help upskill those in industry, ensuring they develop and maintain their analysis skills. Incorporating the data collected by private entities also facilitates collaborations between industry and academia to develop new algorithms and pipelines that are relevant to public health.

Finally, we provide the option for other organizations to deploy analogous versions of HDN that best suit their local needs because all the code for the platform is open source. Likewise, because the code is open source, we welcome collaboration with other organizations to augment the content and delivery of HDN. This is an exciting prospect that could substantially benefit our partners in the future.

## Conclusion

We developed the HDN platform, and through this article speak to the motivation and design principles through to its development and use by the research community. We highlighted how security, scalability, streamlined design, and a social community of open-source developers have led to a successful development, and also how focusing on the education offerings and workshops has spurred interest among users. Ultimately, HDN is positioned to provide a wide variety of multimodal data for educators, trainees, and AI researchers from a variety of backgrounds, with a unique and effective access model. By facilitating access to data, we hope to enrich the medical research ecosystem and aid in the development of patient-centric technologies that will further advance health through AI.

## Supplementary Material

giaf050_Supplemental_Files

giaf050_Authors_Response_To_Reviewer_Comments_original_submission

giaf050_GIGA-D-24-00383_original_submission

giaf050_GIGA-D-24-00383_Revision_1

giaf050_Reviewer_1_Report_original_submission Hollis Lai -- 10/30/2024

giaf050_Reviewer_1_Report_Revision_1 Hollis Lai -- 4/1/2025

## Data Availability

All data referenced in this publication are available open access on HDN [4].

## Additional Files

**Supplementary Fig. S1**. Workflow for data holders to upload data onto the platform.

**Supplementary Fig. S2**. Workflow for users to access data on the platform.

## Abbreviations

AI: artificial intelligence; BSD: Berkeley Software Distribution; CT: computed tomography; FHIR: Fast Healthcare Interoperability Resources; GCP: Google Cloud Platform; GIM: General Internal Medicine; GPU: Graphics Processing Unit; HDN: Health Data Nexus; IRB: institutional review board; ML: machine learning; PHIPA: Personal Health Information Protection Act; PIA: privacy impact assessment; REB: research ethics board; T-CAIREM: Temerty Centre for Artificial Intelligence Research and Education in Medicine; TCPS: Tri-Council Policy Statement; TCPS-CORE: Tri-Council Policy Statement—Course on Research Ethics; TRA: threat risk assessment; VADA: Visual and Automated Disease Analytics.
