# Supplementary figures and images for: Health Data Nexus: an open data platform for AI research and education in medicine
Source: Gigascience. 2025 Jun 3;14:giaf050. doi: 10.1093/gigascience/giaf050 (PMC12131319; doi:10.1093/gigascience/giaf050)

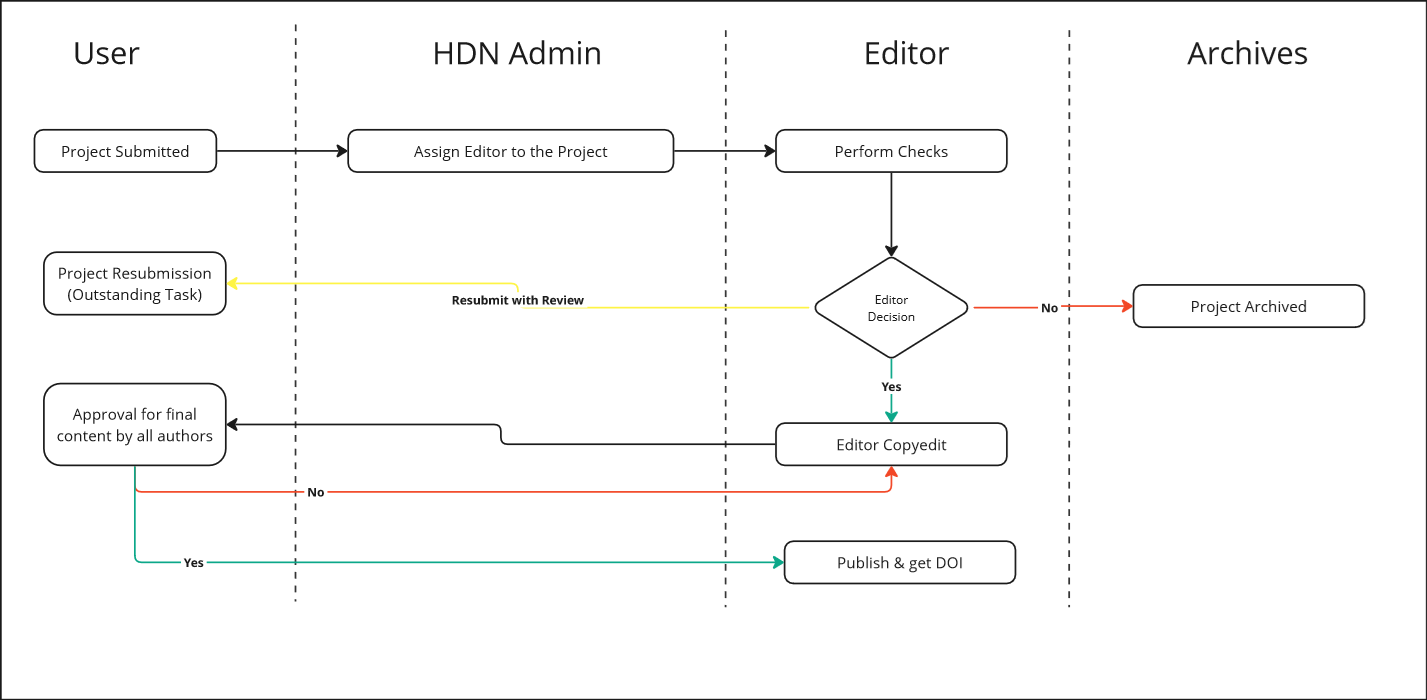

Supplement: giaf050_Supplemental_Files [file giaf050_supplemental_files.zip › supplemental_figure1.png]

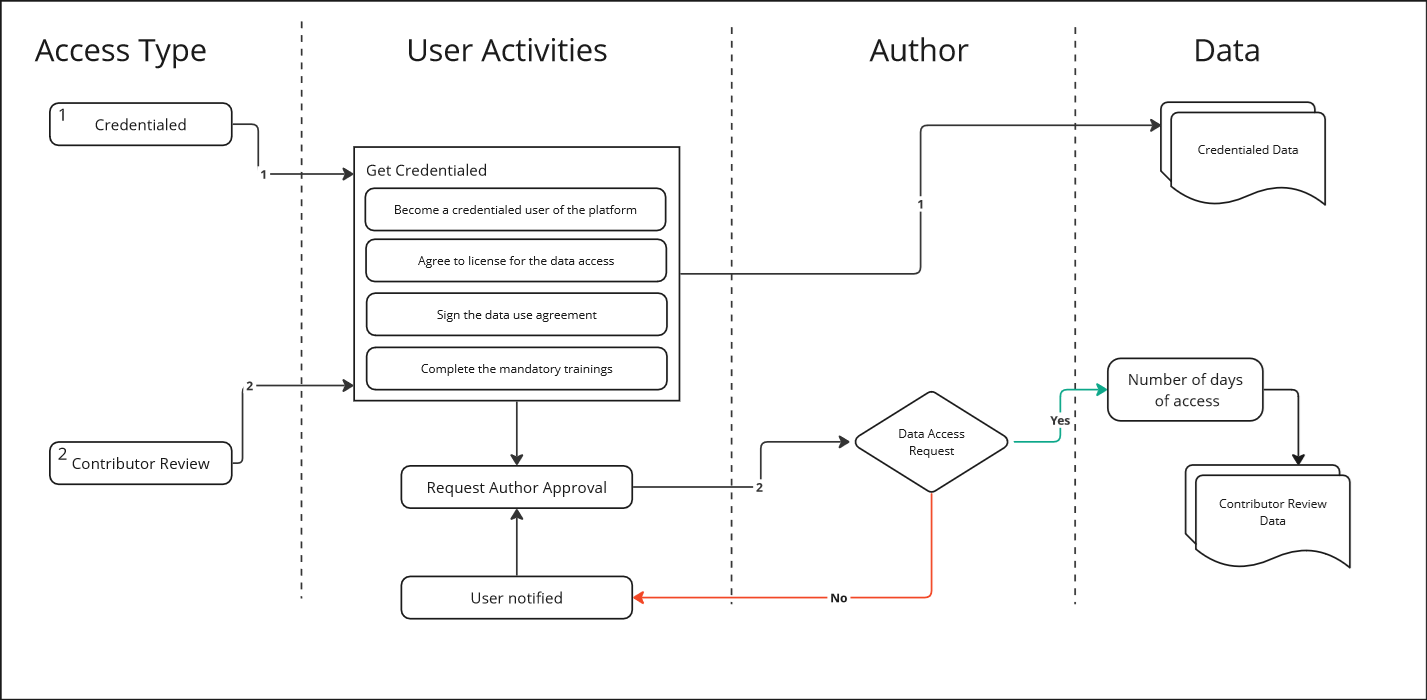

Supplement: giaf050_Supplemental_Files [file giaf050_supplemental_files.zip › supplemental_figure2.png]
