# Supplementary material for: Health Data Nexus: an open data platform for AI research and education in medicine
Source: Gigascience. 2025 Jun 3;14:giaf050. doi: 10.1093/gigascience/giaf050 (PMC12131319; doi:10.1093/gigascience/giaf050)
Supplement: giaf050_Authors_Response_To_Reviewer_Comments_original_submission [file giaf050_authors_response_to_reviewer_comments_original_submission.pdf]

Author's Response To Reviewer Comments

Response to Reviewers' comments

We thank the reviewers for their response and constructive comments for our manuscript titled "Health Data Nexus: An Open Data Platform for AI Research and Education in Medicine" (GIGA-D-24-00383). Based on the reviewers' feedback, we have made substantial changes to the revised manuscript. Please find our response to the reviewers' comments below. Our responses are in italic blue and the reviewers' comments are in black.
Editor
In addition, please register any new software application in the bio.tools and SciCrunch.org databases to receive RRID (Research Resource Identification Initiative ID) and biotoolsID identifiers, and include these in your manuscript. Computational workflows should be registered in workflowhub.eu and the DOIs cited in the relevant places in the manuscript. These will facilitate tracking, reproducibility and re-use of your tool.

We thank the Editor for the recommendations. Health Data Nexus has been registered as an openly available software application for usage in SciCrunch under the id : SCR_026576(pending), and in biotools : hdn (https://bio.tools/hdn)
Reviewer #1
Reviewer #1: The purpose of the paper is to demonstrate the adoption of PhysioNet as a medical data sharing platform. The authors outlined the process, workflow, and approval chain required to facilitate such process. The manuscript also provided initial data use and adoption to demonstrate feasibility of such platform. This is a difficult subject to publish as authors do demonstrate the use of platform, but it is difficult to present this subject in a scientific basis.

We thank the Reviewer for the positive assessment of our manuscript and the constructive comments, which we addressed in details below.

1. The authors describe the datalake require for sharing medical data and does a good job on describing the administrative processes required for such datalake. However, how does this differ from the literature of other platforms? Why was this platform adopted and not other approaches? What information is provided in this adoption that other approaches did not consider or would need to know. I think there is an established literature out there on health data sharing platform that the authors should acknowledge, and highlight how this approach is needed to address these issues.

We thank the reviewer for highlighting the need for more details regarding the uniqueness of the Health Data Nexus platform (HDN). We developed HDN to focus on a unique combination of security, accessibility, and computational scalability. HDN integrates a built-in cloud-based research environment with pre-configured Jupyter and RStudio setups, allowing seamless data analysis without hardware constraints.

A multi-tiered access model (Zones 1–3) ensures secure yet flexible governance, balancing openness with data protection. HDN complies with Canadian privacy laws (PHIPA) and HIPAA, exclusively hosting de-identified health data while leveraging Google Cloud's security to prevent unauthorized data exfiltration.

HDN is uniquely tailored for three user groups:
Data Holders gain secure, cost-effective storage with granular access controls.
Researchers access high-quality datasets with built-in computational tools.
Educators benefit from easy dataset provisioning for workshops, courses, and datathons.

The platform supports multimodal datasets (tabular, imaging, text, and physiological data) and currently hosts seven diverse datasets, with plans to expand into genomics and FHIR-compliant records.

Built on open-source PhysioNet infrastructure, HDN incorporates enhanced scalability, usability, and cloud-native features. Developed through an academic-industry collaboration (University of Toronto, MIT, Upside Labs), it follows Agile development principles, integrating community feedback.

By bridging secure data access, computational efficiency, and education-focused tools, HDN outperforms traditional repositories, fostering scalable AI-driven medical research and innovation.

To emphasize the uniqueness of the Health Data Nexus Platform, we have added a table in the main manuscript (Table 1) comparing our platform to 3 other prominent data platforms. This table is clearly described in the manuscript as being non-exhaustive and we refer to recent review papers on the topic for interested readers.

2. The authors highlight adoption data, but no evaluation data was solicited nor provided. Such information would be helpful to know if we were to evaluate how this creation could be replicated.

We appreciate the reviewer's insight on providing evaluation data. We have included additional information on how the platform has been received by users based on a survey conducted of users at the 2025 Toronto Health Datathon (n=24), which used the Health Data Nexus for data access.

I think there are many great use cases for this outcome but very little is discussed on how it could be applied in the field. For example, is this a method paper promotine others in adopting the platform? or is this a paper demonstrating how others can develop similar platforms?

We thank the reviewer for pointing out that our manuscript can achieve 2 objectives: (1) increase the visibility of the Health Data Nexus platform to attract new users and (2) motivate groups who have to deploy their own data platform to leverage, and potentially contribute, to the code base of Health Data Nexus to adopt it to their needs. We hope that our manuscript will achieve both objectives, using our use cases as inspiration for future use and deployment of similar platforms. Data and methods used to build the platform are open source and available on GitHub.

3. There was acutally no relation to AI other than the use of data holding for AI training. The data holding would make sense for UToronto as the process and approvals are built based on local institution requirements. I have tried to access the system as an external and found it intuitive. But, other than building this platform for the purposes of UToronto holding data for UToronto researchers, is there any plans or process for adopting holdings for other institution? How should other users perceive this information? Could other holdings such as administrative data be used?

We thank the reviewer for their interest in expanding the reach of the Health Data Nexus. The reviewer is right that the Health Data Nexus platform focuses on providing data that are ready for statistical, machine learning and AI analyses. As the platform is used for both education and research, we will attach computer code to the datasets which will include all forms of analyses and AI models, which will greatly augment the value of the data for HDN users. We have clarified this important point in the discussion section of the manuscript.

The platform currently holds data from hospitals and health systems throughout the province of Ontario, including Unity Health, Trillium Health Partners, and Sunnybrook Research Institute, all of which are connected with the University of Toronto through their involvement in the Toronto Academic Health Science Network (TAHSN). In addition, data is also provided from other institutions, such as the Bridge2AI Voice Dataset which includes data from a variety of different cities across Canada and the United States and is based out of the University of South Florida. The Health Data Nexus is open to receiving all forms of data related to the health and medical fields, including administrative data.

I think the presentation of the article has merit but more needs to be done to capture what has already been done in the field and why this solution also needs to be presented (contribution to the field).

We thank the reviewer for all their comments and hope that the responses and updates to the paper adequately address the comments and request for changes.

Cookie Preference Center

We use cookies which are necessary to make our site work. We may also use additional cookies to analyze, improve and personalize our content and your digital experience. For more information, see our Cookie Policy.

You may choose not to allow some types of cookies. However, blocking some types may impact your experience of our site and the services we are able to offer. See the different category headings below to find out more or change your settings.

Allow all

Manage Consent Preferences

Strictly Necessary Cookies

Always active

These cookies are necessary for the website to function and cannot be switched off in our systems. They are usually only set in response to actions made by you which amount to a request for services, such as setting your privacy preferences, logging in or filling in forms. You can set your browser to block or alert you about these cookies, but some parts of the site will not then work. These cookies do not store any personally identifiable information.

Functional Cookies

These cookies enable the website to provide enhanced functionality and personalisation. They may be set by us or by third party providers whose services we have added to our pages. If you do not allow these cookies then some or all of these services may not function properly.

Performance Cookies

These cookies allow us to count visits and traffic sources so we can measure and improve the performance of our site. They help us to know which pages are the most and least popular and see how visitors move around the site.

Targeting Cookies

These cookies may be set through our site by our advertising partners. They may be used by those companies to build a profile of your interests and show you relevant adverts on other sites. If you do not allow these cookies, you will experience less targeted advertising.

Cookie List

Clear

Apply Cancel
Consent Leg.Interest

Confirm my choices

Powered by onetrust
