# Supplementary material for: Health Data Nexus: an open data platform for AI research and education in medicine
Source: Gigascience. 2025 Jun 3;14:giaf050. doi: 10.1093/gigascience/giaf050 (PMC12131319; doi:10.1093/gigascience/giaf050)
Supplement: giaf050_Reviewer_1_Report_original_submission — Hollis Lai -- 10/30/2024 [file giaf050_reviewer_1_report_original_submission.pdf]

Reviewer Report

Title: Health Data Nexus: An Open Data Platform for AI Research and Education in Medicine

Version: Original Submission **Date:** 10/30/2024

Reviewer name: Hollis Lai

Reviewer Comments to Author:

The purpose of the paper is to demonstrate the adoption of PhysioNet as a medical data sharing platform. The authors outlined the process, workflow, and approval chain required to facilitate such process. The manuscript also provided initial data use and adoption to demonstrate feasibility of such platform. This is a difficult subject to publish as authors do demonstrate the use of platform, but it is difficult to present this subject in a scientific basis.1. The authors describe the datalake require for sharing medical data and does a good job on describing the administrative processes required for such datalake. However, how does this differ from the literature of other platforms? Why was this platform adopted and not other approaches? What information is provided in this adoption that other approaches did not consider or would need to know. I think there is an established literature out there on health data sharing platform that the authors should acknowledge, and highlight how this approach is needed to address these issues.2. The authors highlight adoption data, but no evaluation data was solicited nor provided. Such information would be helpful to know if we were to evaluate how this creation could be replicated. I think there are many great use cases for this outcome but very little is discussed on how it could be applied in the field. For example, is this a method paper promotine others in adopting the platform? or is this a paper demonstrating how others can develop similar platforms?3. There was acutally no relation to AI other than the use of data holding for AI training. The data holding would make sense for UToronto as the process and approvals are built based on local institution requirements. I have tried to access the system as an external and found it intuitive. But, other than building this platform for the purposes of UToronto holding data for UToronto researchers, is there any plans or process for adopting holdings for other institution? How should other users perceive this information? Could other holdings such as administrative data be used?I think the presentation of the article has merit but more needs to be done to capture what has already been done in the field and why this solution also needs to be presented (contribution to the field).

Level of Interest

Please indicate how interesting you found the manuscript: Choose an item.

Quality of Written English

Please indicate the quality of language in the manuscript: Choose an item.

Declaration of Competing Interests

Please complete a declaration of competing interests, considering the following questions:

- Have you in the past five years received reimbursements, fees, funding, or salary from an organisation that may in any way gain or lose financially from the publication of this manuscript, either now or in the future?
- Do you hold any stocks or shares in an organisation that may in any way gain or lose financially from the publication of this manuscript, either now or in the future?
- Do you hold or are you currently applying for any patents relating to the content of the manuscript?
- Have you received reimbursements, fees, funding, or salary from an organization that holds or has applied for patents relating to the content of the manuscript?
- Do you have any other financial competing interests?
- Do you have any non-financial competing interests in relation to this paper?

If you can answer no to all of the above, write 'I declare that I have no competing interests' below. If your reply is yes to any, please give details below.

I declare that I have no competing interest.

I agree to the open peer review policy of the journal. I understand that my name will be included on my report to the authors and, if the manuscript is accepted for publication, my named report including any attachments I upload will be posted on the website along with the authors' responses. I agree for my report to be made available under an Open Access Creative Commons CC-BY license (http://creativecommons.org/licenses/by/4.0/). I understand that any comments which I do not wish to be included in my named report can be included as confidential comments to the editors, which will not be published.

Choose an item.

To further support our reviewers, we have joined with Publons, where you can gain additional credit to further highlight your hard work (see: https://publons.com/journal/530/gigascience). On publication of this paper, your review will be automatically added to Publons, you can then choose whether or not to claim your Publons credit. I understand this statement.

Yes Choose an item.
